# Shared HLA Class I and II Alleles and Clonally Restricted Public and Private Brain-Infiltrating αβ T Cells in a Cohort of Rasmussen Encephalitis Surgery Patients

**DOI:** 10.3389/fimmu.2016.00608

**Published:** 2016-12-19

**Authors:** Sugandha Dandekar, Hemani Wijesuriya, Tim Geiger, David Hamm, Gary W. Mathern, Geoffrey C. Owens

**Affiliations:** ^1^Department of Medicine, David Geffen School of Medicine at the University of California Los Angeles, Los Angeles, CA, USA; ^2^Pathology and Laboratory Medicine, David Geffen School of Medicine at the University of California Los Angeles, Los Angeles, CA, USA; ^3^Adaptive Biotechnologies Inc., Seattle, WA, USA; ^4^Department of Neurosurgery, David Geffen School of Medicine at the University of California Los Angeles, Los Angeles, CA, USA; ^5^Intellectual and Developmental Disabilities Research Center, David Geffen School of Medicine at the University of California Los Angeles, Los Angeles, CA, USA; ^6^Brain Research Institute, David Geffen School of Medicine at the University of California Los Angeles, Los Angeles, CA, USA; ^7^Mattel Children’s Hospital, Los Angeles, CA, USA

**Keywords:** Rasmussen encephalitis, T cell receptor, human leukocyte antigen, epilepsy, autoimmunity

## Abstract

Rasmussen encephalitis (RE) is a rare pediatric neuroinflammatory disease characterized by intractable seizures and unilateral brain atrophy. T cell infiltrates in affected brain tissue and the presence of circulating autoantibodies in some RE patients have indicated that RE may be an autoimmune disease. The strongest genetic links to autoimmunity reside in the MHC locus, therefore, we determined the human leukocyte antigen (HLA) class I and class II alleles carried by a cohort of 24 RE surgery cases by targeted in-depth genomic sequencing. Compared with a reference population the allelic frequency of three alleles, DQA1*04:01:01, DQB1*04:02:01, and HLA-C*07:02:01:01 indicated that they might confer susceptibility to the disease. It has been reported that HLA-C*07:02 is a risk factor for Graves disease. Further, eight patients in the study cohort carried HLA-A*03:01:01:01, which has been linked to susceptibility to multiple sclerosis. Four patients carried a combination of three HLA class II alleles that has been linked to type 1 diabetes (DQA1*05:01:01:01~DQB1*02:01:01~DRB1*03:01:01:01), and five patients carried a combination of HLA class II alleles that has been linked to the risk of contracting multiple sclerosis (DQA1*01:02:01:01, DQB1*06:02:01, DRB1*15:01:01:01). We also analyzed the diversity of αβ T cells in brain and blood specimens from 14 of these RE surgery cases by sequencing the third complementarity regions (CDR3s) of rearranged T cell receptor β genes. A total of 31 unique CDR3 sequences accounted for the top 5% of all CDR3 sequences in the 14 brain specimens. Thirteen of these sequences were found in sequencing data from healthy blood donors; the remaining 18 sequences were patient specific. These observations provide evidence for the clonal expansion of public and private T cells in the brain, which might be influenced by the RE patient’s HLA haplotype.

## Introduction

Rasmussen encephalitis (RE) is a very rare neurological disorder of unknown etiology for which there is no known cure. The disease primarily affects young children starting around 6 years of age (range 1–14 years), and if untreated results in permanent deficits affecting motor and sensory systems (1–3). In general patients first present with partial (focal) seizures that are refractory to antiepileptic drug therapy; seizures may spread to ipsilateral brain seriously impacting intellectual development. Magnetic resonance images show areas of inflammation and atrophy usually in one cerebral hemisphere (1). Immunosuppressive therapies have been partially effective (4–8), although surgery may ultimately be the only treatment option that can control the intractable seizures.

Examination of resected brain tissue indicates that the inflammatory reaction in RE involves activated brain resident macrophages (microglia) and both αβ and γδ T cells (9–13). CD8^+^ T cells with polarized granzyme B-containing granules have been observed in sections of resected brain tissue in close apposition to both neurons and astrocytes (9, 14), suggesting that they may have formed immunological synapses and may be engaged in MHC class I-restricted cell killing. By contrast, cytotoxic γδ T cells do not depend on antigen presentation by classical MHC molecules (15–17). Clonal expansion of αβ T cells in RE brain tissue and in blood has been documented providing evidence for an antigen-driven immune response in RE (18, 19). More recently, deep sequencing the third complementarity regions (CDR3s) of the T cell receptor (TCR) β locus from cDNA prepared from CD8^+^ peripheral blood mononuclear cells (PBMCs) from 23 RE cases and from five RE brain tissue specimens has confirmed this observation and has identified potentially RE-specific T cell clones (20). Similarly, we have shown that Vδ1^+^ γδ T cells in RE brain tissue are clonally restricted (13).

To gain further insight into the involvement of αβ T cells in RE, we carried out deep sequencing of rearranged TCR β CDR3s from genomic DNA isolated from matched blood and brain tissue from 14 RE surgery cases. By comparing these sequences to blood from 14 other pediatric epilepsy surgery cases and a cohort of 100 normal individuals, we identified clonally expanded private (patient-specific) and public T cells in RE brain tissue.

Several alleles of classical human leukocyte antigen (HLA) genes in the MHC locus have been linked to autoimmune diseases. We sequenced the major HLA I and II genes in 24 RE cases and found that 16 of these RE cases carried HLA I and/or II alleles that have been linked to the risk of contracting an autoimmune disease.

## Materials and Methods

All of the surgical specimens used in this study were obtained under IRB approval (UCLA IRB nos. 11-00030 and 13-001213) with informed consent and in compliance with HIPAA requirements, as part of a multi-institutional RE tissue transfer program (21). All of the RE patients in the study cohort were Caucasian with a median age of 8.5 years (range 3–14.4 years) at the time of surgery. All specimens and patient data were de-identified, and there were no exclusion criteria. Treatment histories prior to surgery were not available for all of the RE cases. Genomic DNA was prepared from flash-frozen blocks of involved tissue and from whole blood, collected at the time of surgery, using a DNAeasy blood and tissue kit (Qiagen, Valencia, CA, USA). Access to a database of TCR β CDR3 sequences from anonymized healthy control blood samples was provided by Adaptive Biotechnologies (Seattle, WA, USA).

### HLA Sequencing

A TrueSight^®^ HLA sequencing panel (Illumina, San Diego, CA, USA) was used to obtain in-depth sequence information about the eight major HLA genes (HLA-A, B, C, DRPA1, DRPB1, DRQA1, DRQB1, DRB1/3/4/5) in 24 RE genomic DNA samples. Barcoded libraries were sequenced on a MiSeq platform (Illumina), and FASTQ files were uploaded to BaseSpace (Illumina). Assignments of HLA alleles were made using ConnexioAssign™ v1.0 TrueSight^®^ HLA analysis software (Illumina).

### TCR Vβ Sequencing

An ImmunoSEQ kit (Adaptive Biotechnologies) was used to generate libraries of Vβ CDR3 sequences from 42 genomic DNA samples. The kit incorporates a two-step amplification bias-controlled PCR approach and barcoded spiked-in synthetic templates in order to measure the degree of sequencing coverage and residual PCR bias (22, 23). The CDR3 libraries were sequenced on a MiSeq platform (Illumina), and FASTQ files were uploaded to BaseSpace (Illumina) and curated at Adaptive Biotechnologies. To merge closely related sequences, the data were filtered and clustered using both the relative frequency ratio between similar clones and a modified nearest-neighbor algorithm. The resulting sequences were annotated according to the IMGT database^1^ (24). The TCR sequences were normalized to correct for residual multiplex PCR amplification bias and quantified against a set of synthetic TCR β CDR3 sequences. The data were further curated *via* the IMGT HighV-QUEST web portal to remove all non-productive rearrangements (25). Clonality was calculated as described by Harden et al. (26). Graphs and statistical analysis utilized Microsoft Excel^®^ (Microsoft Corporation, Redmond, WA, USA) and R-project programs.^2^ Curve fitting utilized EasyFit5.6 Professional,^3^ relative risks were determined using an online calculator,^4^ Venn diagrams were constructed using online software,^5^ and the heat map was generated with GENE-E bioinformatics software tools.^6^ Plots were exported to CorelDRAW X6 (Corel Corporation, Ottawa, ON, Canada).

## Results

### RE Patients Carry Autoimmune Disease-Associated HLA Alleles

We obtained sequence information for the major HLA genes from 24 RE cases (Table S1 in Supplementary Material). Strikingly, two unrelated patients, RECP43 and RECP48, had the identical HLA haplotype (Table S1 in Supplementary Material). Table 1 lists the alleles that were shared by four or more cases; DPA1*01:03:01:01 was the most frequent allele, but this may be expected from the demographics of our patient cohort; in a study of 5,944 stem cell donors self-identified as European Caucasian, the allelic frequency of DPA1*01:03 was 0.8186 (27). The allelic frequencies for all but three of the overlapping HLA alleles have been reported for a population of 220 Caucasians from the San Francisco Bay Area^7^ (28, 29), which allowed us to calculate provisional relative risks. Compared with this reference population, three alleles were associated with a statistically significant (*P* < 0.05) increased relative risk of RE, C*07:02:01:01, DQA1*04:01:01, and DQB1*04:02:01 (Table 1). Six patients carried both DQA1*04:01:01, and DQB1*04:02:01 suggesting that these gene products form a heterodimer. It will be necessary to screen a larger cohort of RE patients to substantiate the risk alleles that we have identified.

**Table 1:** **Frequency of human leukocyte antigen alleles in a cohort of Rasmussen encephalitis patients**.

Allele	Allele frequency	Allele frequency (USA, a Caucasian population)	Relative risk	95% CI	*P*-value*
A*02:01:01:01	0.229	0.242	0.9424	0.5468–1.6242	0.84186
A*03:01:01:01	0.167	0.148	1.1282	0.5766–2.2075	0.73775
A*24:02:01:01	0.104	0.75	1.3889	0.5691–3.3895	0.47993
A*29:02:01:01	0.104	0.48^a^	2.1825	0.8622–5.5245	0.09930
B*07:02:01	20.8	0.124	1.6667	0.9107–3.0501	0.09733
B*44:03:01:01	0.104	0.053^b^	1.9928	0.7941–5.0009	0.14216
C*04:01:01:01	0.083	0.102	0.8148	0.3063–2.1673	0.69472
C*05:01:01:01	0.083	0.076	1.1111	0.4112–3.0023	0.84629
C*07:02:01:01	0.25	0.141	1.7742	1.0323–3.0494	0.03767
DPA1*01:03:01:01	0.75	Not reported			
DPA1*02:02:02	0.083	Not reported			
DPB1*01:01:01	0.125	0.057^c^	2.2	0.95–5.0947	0.06533
DPB1*04:01:01:01	0.375	0.407^d^	1.257	0.8551–1.8478	0.24719
DPB1*04:02:01:01	0.125	0.105^e^	1.1957	0.5389–2.6527	0.67329
DQA1*02:01	0.146	0.118^f^	1.234	0.5943–2.5624	0.58465
DQA1*01:02:01:01	0.167	0.225	0.7407	0.3844–1.4274	0.37619
DQA1*03:01:01	0.125	0.159	0.7857	0.3606–1.7118	0.55516
DQA1*04:01:01	0.146	0.034	4.5833	1.9453–10.786	0.00053
DQA1*05:01:01:01	0.083	0.261^g^	0.3188	0.1231–0.8255	0.01840
DQA1*05:05:01:01	0.125	Not reported			
DQB1*02:01:01	0.083	0.153	0.5473	0.2087–1.4348	0.22232
DQB1*02:02:01	0.083	0.089^h^	0.9402	0.3511–2.5175	0.91006
DQB1*03:01:01:01	0.146	0.158^i^	0.93	0.4535–1.9070	0.85357
DQB1*04:02:01	0.167	0.027^j^	6.1111	2.6289–14.2059	<0.0001
DQB1*06:02:01	0.104	0.155	0.674	0.2858–1.5895	0.37375
DRB1*03:01:01:01	0.083	0.145	0.5729	0.2182–1.5041	0.26101
DRB1*07:01:01:01	0.146	0.12	1.2107	0.5836–2.5116	0.62007
DRB1*15:01:01:01	0.146	0.159	0.9167	0.4473–1.8787	0.82381

*P values are calculated from 95% confidence intervals according to Altman and Bland (30).

^a^A*29:02:01.

^b^B*44:03:01.

^c^DPB1*01:01.

^d^DPB1*04:01:01.

^e^DPB1*04:02.

^f^DQA1*01:02:01.

^g^DQA1*05:01:01.

^h^DQB1*02:02.

^i^DQB1*03:01:01.

^j^DQB1*04:02:01.

It is interesting to note that HLA-C*07 has been reported to be a risk factor for Graves disease, an autoimmune disease that affects the thyroid gland (31). Of the 10 RE cases that carried HLA-C*07:02:01:01, six of them were also positive for HLA-A*03:01:01:01, which has been linked to MS (32) (Table 2). HLA class II alleles found in our patient cohort have also been associated with autoimmunity. As shown in Table 3, four RE patients were positive for the DRB1*03:01-DQA1*05:01-DQB1*02:01 haplotype (DR3 serotype) that confers susceptibility to type 1 diabetes (33), likewise the haplotype DRB1*15:01-DQA1*01:02-DQB1*0602 increases the risk of contracting MS (32), and five different RE patients carried all three alleles (Table 3). These data suggest that some RE cases might be associated with an autoimmune response.

**Table 2:** **Rasmussen encephalitis cases in the study cohort carrying human leukocyte antigen (HLA) class I alleles that have been linked to an autoimmune disease**.

	HLA A		HLA B		HLA C	
Case ID	Allele 1	Allele 2	Allele 1	Allele 2	Allele 1	Allele 2
RECP03	A*03:01:01:01	A*32:01:01	B*44:02:01:01	B*40:02:01	C*03:04:01:01	C*05:01:01:01
RECP12	A*03:01:01:01	A*33:01:01	B*07:02:01	B*27:03	C*07:02:01:01	C*02:02:02:01
RECP16	A*03:01:01:01	A*24:02:01:01	B*14:02:01:01	B*39:06:02	C*07:02:01:01	C*08:02:01:01
RECP26	A*03:01:01:01	A*24:02:01:01	B*07:02:01	B*27:05:02	C*07:02:01:01	C*01:02:01
RECP27	A*03:01:01:01	A*02:01:01:01	B*07:02:01	B*39:06:02	C*07:02:01:01	C*07:02:01:01
RECP34	A*03:01:01:01	A*24:02:01:01	B*07:02:01	B*13:02:01	C*07:02:01:01	C*06:02:01:01
RECP39	A*03:01:01:01	A*02:01:01:01	B*07:02:01	B*07:02:01	C*07:02:01:01	C*07:02:01:01
RECP40	A*68:01:02:01	A*68:01:02:01	B*07:02:01	B*40:01:02	C*07:02:01:01	C*03:04:01:01
RECP42	A*11:01:01:01	A*23:01:01	B*07:02:01	B*07:05:01	C*07:02:01:01	C*15:05:02
RECP45	A*24:02:01:01	A*02:01:01:01	B*15:01:01:01	B*39:06:02	C*07:02:01:01	C*03:03:01
RECP49	A*03:01:01:01	A*29:02:01:01	B*07:02:01	B*14:02:01:01	C*07:02:01:01	C*08:02:01:01

*RECP, Rasmussen Encephalitis Children’s Project*.

**Table 3:** **Rasmussen encephalitis cases in the study cohort carrying HLA class II alleles that have been linked to an autoimmune disease**.

	DQA1		DQB1		DRB1	
Case ID	Allele 1	Allele 2	Allele 1	Allele 2	Allele 1	Allele 2
RECP02	DQA1*05:01:01:01	DQA1*02:01	DQB1*02:01:01	DQB1*02:02:01:01	DRB1*03:01:01:01	DRB1*07:01:01:01
RECP32	DQA1*05:01:01:01	DQA1*04:01:01	DQB1*02:01:01	DQB1*04:02:01	DRB1*03:01:01:01	DRB1*03:02:01
RECP33	DQA1*05:01:01:01	DQA1*05:05:01:01	DQB1*02:01:01	DQB1*03:19	DRB1*03:01:01:01	DRB1*11:02:01
RECP37	DQA1*05:01:01:01	DQA1*01:03:01:01	DQB1*02:01:01	DQB1*06:03:01	DRB1*03:01:01:01	DRB1*13:01:01
RECP26	DQA1*01:02:01:01	DQA1*05:05:01:01	DQB1*06:02:01	DQB1*03:01:01:01	DRB1*15:01:01:01	DRB1*01:03
RECP39	DQA1*01:02:01:01	DQA1*03:02	DQB1*06:02:01	DQB1*03:03:02:01	DRB1*15:01:01:01	DRB1*09:01:02
RECP42	DQA1*01:02:01:01	DQA1*02:01	DQB1*06:02:01	DQB1*02:02:01:01	DRB1*15:01:01:01	DRB1*07:01:01:01
RECP49	DQA1*01:02:01:01	DQA1*01:02:01:01	DQB1*06:02:01	DQB1*06:09:01	DRB1*15:01:01:01	DRB1*13:02:01
RECP50	DQA1*01:02:01:01	DQA1*02:01	DQB1*06:02:01	DQB1*02:02:01	DRB1*15:01:01:01	DRB1*07:01:01:01

*RECP, Rasmussen Encephalitis Children’s Project*.

### Higher TCR Clonality in Brain versus Matched Blood

**Figure 1:**
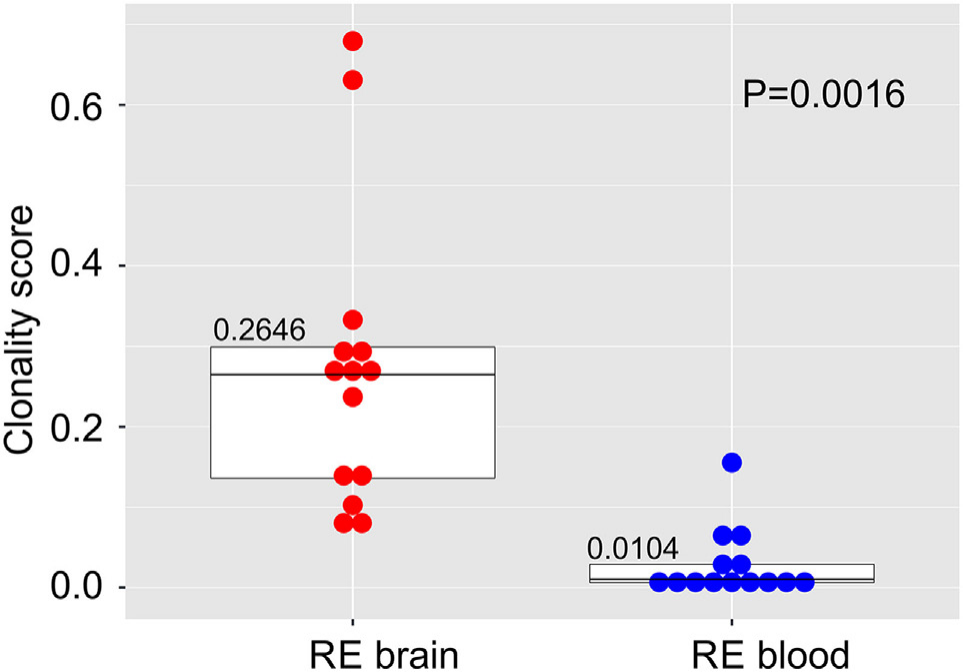
**Clonal restriction of αβ T cells in resected brain tissue from surgeries to treat Rasmussen encephalitis compared with matched blood samples collected at the time of surgery**. The frequencies of unique CDR3 amino acid sequences in each brain and blood specimen were used to calculate a clonality score. Stacked dot/box plots with median clonality scores show that the repertoire of T cells in the brain is significantly less diverse than in the circulation (*N* = 14 patients). *P* value was determined by the Wilcoxon signed-rank test.

**Figure 2:**
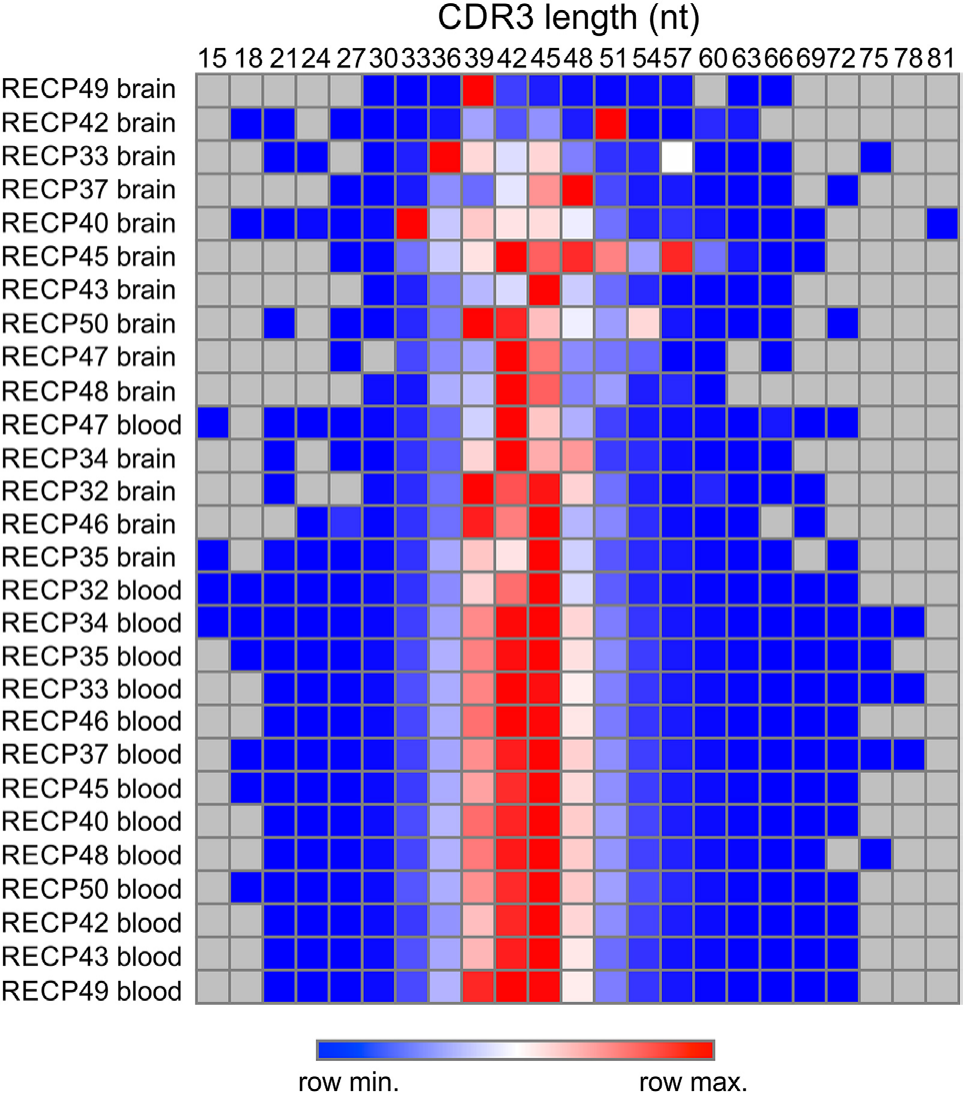
**Length distribution of CDR3 sequences in Rasmussen encephalitis brain and blood samples**. The lengths in nucleotides of all of the CDR3 sequences in brain and matched blood specimens were compiled, and the frequency distributions were used to generate a heat map.

We compared the TCR β CDR3 amino acid sequences in brain and matched blood samples from 14 RE surgeries. To obtain an overall measure of the diversity of the CDR3 sequences in each sample, a clonality score was calculated. Clonality is defined as 1 minus the normalized Shannon’s Diversity Index, and varies from 0, maximal diversity, to 1 in a completely oligoclonal sample. Evenness metrics such as clonality allow comparisons to be made between samples that vary in sequencing depth (26). As shown in Figure 1 the diversity of CDR3 sequences in the brain was significantly less than in the blood, although the clonality of the brain specimens varied, and the clonal composition of one of the blood samples was clearly skewed. To further explore the differences between the brain and blood TCR repertoires, we analyzed the length distribution of nucleotide sequences that specified each CDR3 amino acid sequence. In Figure 2, these distributions are represented as a heat map following Euclidean clustering. All of the brain samples and all except one of the blood samples are clustered. The outlier, RECP47 corresponds to the blood sample with the high clonality score (Figure 1). The CDR3 length distributions in the blood were centered at 42 and 45 nucleotides whereas the CDR3 length distributions in the brain appeared to be more variable. Five of the brain samples peaked at shorter (RECP33, RECP40, and RECP49) or longer (RECP37 and RECP42) nucleotide sequence lengths; RECP45 contained an additional peak at 57 nucleotides. It has been documented that CDR3 length distributions from healthy individuals follow a Gaussian-like distribution (34). We fitted all of the CDR3 distributions to a normal curve and applied the Kolmogorov–Smirnov statistic to assess Goodness-of-Fit. The RECP49 brain sample was the only case that deviated significantly from a Gaussian distribution according to this analysis, although RECP42 approached significance (Figure S1 in Supplementary Material). However, in four cases (RECP33, RECP35, RECP42, and RECP49), the relative frequency of Vβ genes significantly differed between brain and blood (Figure S2 in Supplementary Material). This could be attributed to the overrepresentation of one particular Vβ gene in the RE brain samples, and may reflect the presence of one or more highly abundant T cell clone in the brain.

### Overlap of CDR3 Sequences between Brain and Blood

**Figure 3:**
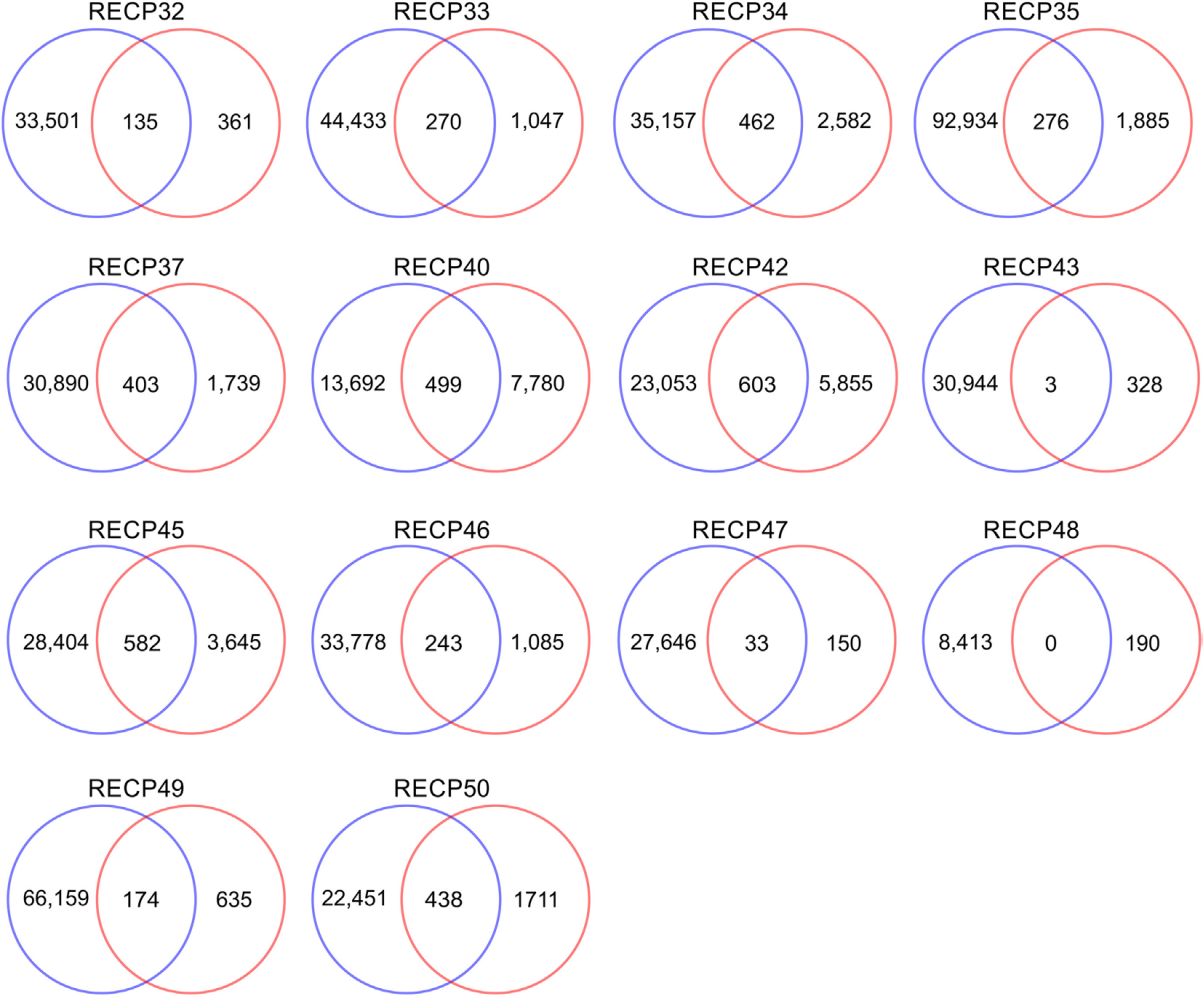
**CDR3 sequence overlap between matched brain and blood specimens**. All of the different CDR3 sequences in brain and matched blood specimens were counted. The results are displayed as Venn diagrams. Average overlap is 14.7% of the brain repertoire.

**Figure 4:**
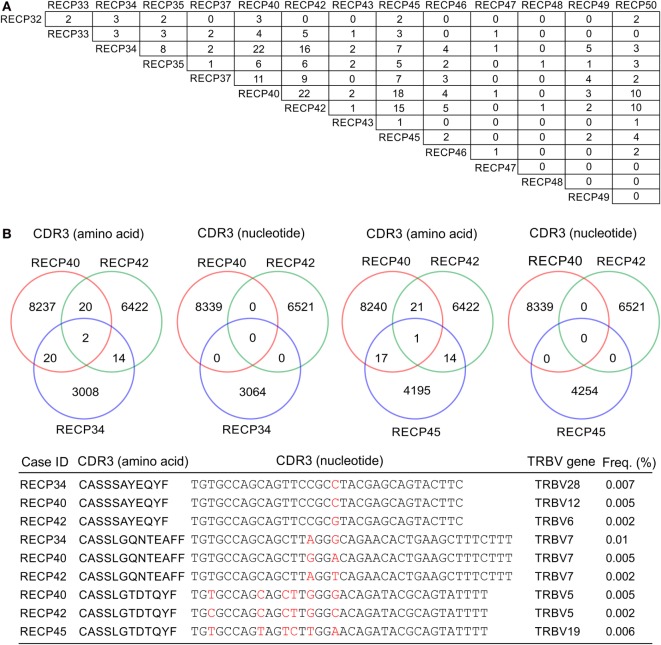
**CDR3 sequence overlap between RE brain specimens**. **(A)** Pairwise comparison of CDR3 sequences from 14 RE brain specimens showing the number of sequences found in more than one brain specimen. **(B)** Cross comparison between cases with the highest number of shared sequences (RECP34, RECP40, RECP42, and RECP45). Venn diagrams show that there is no overlap at the nucleotide level. Nucleotide differences are shown in red.

A pairwise comparison of the CDR3 amino acid sequences in each brain and matched blood sample revealed that there were both shared sequences and sequences that were only found in the brain (Figure 3). In the case of RECP48 there was no overlap likely due to the paucity of CDR3 sequences in the brain tissue specimen. Comparison of the nucleotide sequences encoding the shared CDR3 sequences indicated that some of the T cells in the brain and the blood that expressed the same CDR3 amino acid sequence originated from different clonal lineages (Figure S3 in Supplementary Material). We next determined if any of the CDR3 amino acid sequences in the brain samples were found in more than one brain sample, which proved to be the case (Figure 4A). Comparing the sequences in four brain samples that had the highest number of shared sequences seen in a pair-wise comparison identified three overlapping sequences (Figure 4B). As expected they were encoded by different nucleotide sequences. In the paper by Schneider-Hohendorf et al. the CDR3 sequence CASSLGTDTQYF shared by RECP40, RECP42 and RECP45 was also found in CD8^+^ PBMCs from five RE patients (20). RECP40, RECP42 and RECP45 all carry the HLA class I allele C*07:01:01:01 (Table S1 in Supplementary Material). We looked for all of the sequences that were present in three or more brain samples and found 15 more CDR3 sequences. We compared them with the sequences in the RE blood samples, and in reference groups of blood from 12 focal cortical dysplasia (FCD) cases and two tuberous sclerosis complex (TSC) cases, and 100 healthy individuals. As shown in Table 4, none of the 18 CDR3 sequences were unique to the RE brain samples, which means that there were public T cell clones in the RE brain samples. Strikingly 12 of these sequences were found in >90% of the cohort of healthy individuals, indicating that they correspond to very common T cell clones. Two pairs of the CDR3 sequences differ by only one amino acid suggesting that each pair may recognize the same epitope (Table 4).

**Table 4:** **Incidence of overlapping CDR3 sequences**.

CDR3 (amino acid)	RE brain (%)	RE blood (%)	FCD/TSC blood (%)	Normal blood (%)
CASSLGTDTQYF	36	64	71	99
CASSLSTDTQYF	29	50	57	100
CASSLGETQYF	29	93	79	100
CASSLGGTEAFF	21	71	64	100
CASSSQETQYF	21	57	79	100
CASSLGYEQYF	21	79	79	99
CASSPGYEQYF	21	29	64	92
CASSLGQNTEAFF	21	86	64	100
CASSIQGNTEAFF	21	7	21	69
CASSPLGSSYNEQFF	21	0	7	46
CASSLGQGNTGELFF	21	36	36	83
CASSPRDSYEQYF	21	21	29	68
CASRLGGGTEAFF	21	0	14	19
CASSFYNEQFF	21	29	29	92
CASSPNTEAFF	21	50	57	93
CASSPSGSSYEQYF	21	29	7	73
CASSLAGGTDTQYF	21	57	71	99
CASSSAYEQYF	21	36	64	96

*CDR3, third complementarity region; RE, Rasmussen encephalitis; FCD, focal cortical dysplasia; TSC, tuberous sclerosis complex*.

### Abundant Private and Public T Cell Clones in RE Brain Tissue

The high clonality scores, and skewing of Vβ gene usage and CDR length distributions clearly indicated that there were a number of abundant T cell clones in the RE brain samples. In Table 5 the CDR3 sequences comprising the top ~5% of CDR3 sequences in each brain sample are tabulated; none of the sequences are shared. These sequences appear to account in large measure for the skewing of the distributions of Vβ gene usage and CDR3 length. Several of the sequences were also found in the matched blood sample albeit at a much lower frequency, but with one exception. The top sequence in RECP47 brain is also the top sequence in RECP47 blood, and its frequency in the blood exceeds that of the brain. This explains the outlier in the plot of clonality scores of the blood samples shown in Figure 1. Similarly the two brain samples with the highest clonality scores correspond to RECP42 and RECP49 in which 47and 70%, respectively, of the CDR3 sequences are identical. Nineteen of the CDR3 sequences in the brain samples are associated with more than one TRBV gene (Table 5). However, except for one of the two CDR3 sequences in RECP40, one Vβ gene is associated with 97–99% of the CDR3 sequence (Table S2 in Supplementary Material). In the RECP40 brain the top two CDR3 sequences only differ by one amino acid suggesting that the same epitope may be recognized (Table 5).

**Table 5:** **Top CDR3 sequences in Rasmussen encephalitis brain specimens from the study cohort**.

Case ID	CDR3 (amino acid)	CDR3 length (nt)	Brain (%)	TRBV gene	Blood (%)	TRBV gene
RECP32	CASSQDEGDEQYF	39	9.22	3	0	n.a.
	CASSLNPDRGIYEQFF	48	5.01	7	0	n.a.
RECP33	CAISESNYGYTF	36	22.26	6,10	0.13	6,10
	CASSLLVVESELHTGELFF	57	13.47	4,5	0.004	5
	CASSDSRGNIQYF	39	6.08	6	0	n.a.
	CASSKTSGPDNEQFF	45	5.40	7,11	0.004	7
RECP34	CASAEEWSSYNSPLHF	48	6.65	6,10	0	n.a.
	CASTLLRDTDTQYF	42	4.89	2	0.027	2
	CASSQDTPGQFYEQYF	48	4.62	1,4,5,7,13	0	n.a.
RECP35	CASSLRGTGNTEAFF	45	8.07	2,4,7,12,19,27,28,30	0	n.a.
	CASGPGGPSTGELFF	45	7.18	12,13	0.003	13
	CASSTSSTDTQYF	39	5.15	2,6	0	n.a.
RECP37	CASSQDPQGALNEQFF	48	17.13	4,5,7,11	0.009	4
	CASSYRPESYNEQFF	45	6.83	6	0.044	6
	CASGRGTSGPTGELFF	48	4.95	12	0.003	12
RECP40	CASSVAYEQYF	33	12.31	2,3,6,10,12,25,27	0.006	6
	CASAVAYEQYF	33	9.85	3,6,10,12,27	0	n.a.
RECP42	CASSVDHRAGKPYEQYF	51	47.03	5,19	0.008	19
	CATSVTTGGYTEAFF	45	9.61	12,24	0.059	24
	CASSGGSTDTQYF	39	8.75	4,19	0.004	19
RECP43	CASSTPGQGIGGYTF	45	15.44	19	0	n.a.
	CASSLQDRGPGGEQYV	48	5.65	7,11	0	n.a.
RECP45	CASSLRNYDDRVGYYEQYF	57	13.38	6,12,27,28	0.003	27
	CASSLGTGDRSNQPQHF	51	6.94	6,28	0.009	28
RECP46	CASTLQMNTEAFF	39	5.62	6	0.005	6
RECP47	CASGYEGGSTEAFF	42	9.60	12	15.09	7,12
RECP48	CASSSDGNTGELFF	42	7.49	5	0	n.a.
RECP49	CASQLGAATGYTF	39	70.02	4,5,7,11	3.8	3,4,5,6,7,11
RECP50	CASSPDRVETQYF	39	12.15	7	0	n.a.
	CVSSPGVPFTRFNTEAFF	54	8.40	12	0	n.a.
	CASSLSSFQETQYF	42	5.14	7,11	0	n.a.

*CDR3, third complementarity region; RECP, Rasmussen Encephalitis Children’s Project; n.a., not applicable*.

**Figure 5:**
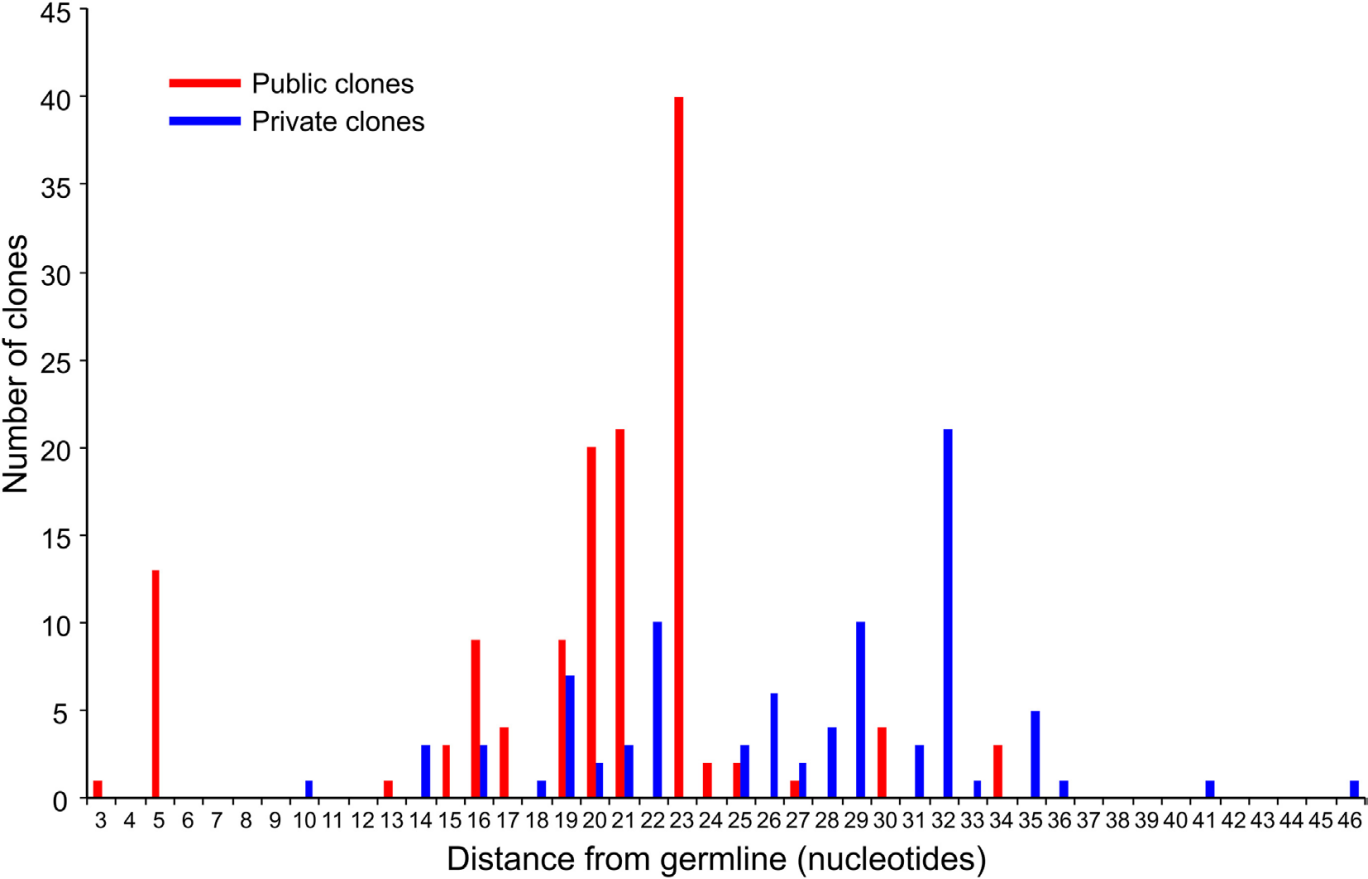
**Distance from germline of the most prevalent private and public clones in RE brain tissue**. The top five percent of CDR3 amino acid sequences in the RE brain samples comprise 133 public and 88 private TCR clones (based on Vβ Dβ, and Jβ usage and the number of nucleotide insertions and deletions at the Vβ/Dβ and Dβ/Jβ boundaries). Summing the number of nucleotide insertions and deletions at the Vβ/Dβ and Dβ/Jβ boundaries provides a measure of the distance from germline of the clone; a larger number reflects a greater distance. The difference between the private and public clones was statistically significant (*P* = < 0.0001, Mann–Whitney test).

We compared the 31 CDR3 sequences that comprised the top ~5% of sequences in the RE brain samples with our sequence data from the 28 RE and FCD/TSC blood samples as well as the cohort of 100 healthy blood donors. As shown in Table 6 18 of the sequences were only found in the brain or in the matched blood sample; however, the remaining CDR3 sequences were not specific to RE. Based on the number of nucleotide insertions and deletions at the VβDβ and DβJβ boundaries, the distance of the private (patient-specific) TCRβ CDR3 sequences from germline was greater than that of the public TCRβ CDR3 sequences (Figure 5). It has been reported that the CDR3 sequences in CD8^+^ T cell clones that are shared between individuals contain fewer nucleotide insertions (35). Two of the public CDR3 sequences in two RE cases differed by a single amino acid from the CDR3 sequences found in two cytotoxic T cell clones that recognize an influenza A virus epitope (36) (Table 7). Both of the clones directed against the influenza virus were HLA-A*02:01-positive whereas neither RE case carried this allele (Table 7). However, the two HLA-A alleles carried by the RE patients are associated with HLA supertypes that are known to bind influenza virus peptides (37, 38).

**Table 6:** **Incidence of public CDR3 sequences**.

CDR3 (amino acid)	RE blood (%)	FCD/TSC blood (%)	Normal blood (%)
CASSGGSTDTQYF	43	14	69
CASSVAYEQYF	14	14	43
CASSPDRVETQYF	0	7	38
CASSTSSTDTQYF	0	14	25
CASSSDGNTGELFF	0	0	21
CASSLRGTGNTEAFF	7	0	17
CAISESNYGYTF	7	7	16
CASSLSSFQETQYF	0	0	6
CASTLQMNTEAFF	7	0	5
CASSQDEGDEQYF	0	7	2
CASAEEWSSYNSPLHF	0	7	2
CASSTPGQGIGGYTF	0	0	2
CASAVAYEQYF	0	0	1
CASSLLVVESELHTGELFF	7	0	0
CASSKTSGPDNEQFF	7	0	0
CASTLLRDTDTQYF	7	0	0
CASGPGGPSTGELFF	7	0	0
CASSQDPQGALNEQFF	7	0	0
CASSYRPESYNEQFF	7	0	0
CASGRGTSGPTGELFF	7	0	0
CASSVDHRAGKPYEQYF	7	0	0
CATSVTTGGYTEAFF	7	0	0
CASSLRNYDDRVGYYEQYF	7	0	0
CASSLGTGDRSNQPQHF	7	0	0
CASGYEGGSTEAFF	7	0	0
CASQLGAATGYTF	7	0	0
CVSSPGVPFTRFNTEAFF	0	0	0
CASSLNPDRGIYEQFF	0	0	0
CASSDSRGNIQYF	0	0	0
CASSQDTPGQFYEQYF	0	0	0
CASSLQDRGPGGEQYV	0	0	0

*CDR3, third complementarity region; RE, Rasmussen encephalitis; FCD, focal cortical dysplasia; TSC, tuberous sclerosis complex*.

**Table 7:** **CDR3 sequences in Rasmussen encephalitis brain that closely match influenza A virus-specific CDR3 sequences**.

ID	CDR3	Human leukocyte antigen-A allele
RECP35	CASST 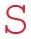 STDTQYF	A*29:02:01:01
Z35705	CASST 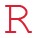 STDTQYF	A*02:01
RECP42	CASSG 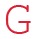 STDTQYF	A*11:01:01:01
Z35681	CASSG 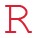 STDTQYF	A*02:01

*IDs: RECP, Rasmussen Encephalitis Children’s, Genbank accession number*.

*CDR3, third complementarity region*.

## Discussion

We and others have reported that the majority of αβ T cells in affected brain parenchyma from RE surgeries are CD8^+^ (9, 10, 13), thus finding a number of shared HLA class I alleles among the 24 RE cases surveyed supports the involvement of antigen specific MHC class I-restricted T cells in RE. Two of the shared HLA class I alleles, HLA-C*03:01:01:01 and HLA-C*07:01:01:01 may predispose an individual to an autoimmune response. It has been reported that HLA-A*03:01 confers susceptibility to MS independent of HLA class II risk alleles (39); the HLA-C*03:01:01:01 allele is carried by a third of the RE cases in our study cohort. HLA-C*07 has been linked to Graves disease, an autoimmune disorder that causes hyperthyroidism (31); 10 of the RE cases carry HLA-C*07:01:01:01, and six patients carry both HLA-C*03:01:01:01 and HLA-C*07:01:01:01. Sixteen of the RE cases reported by Schneider-Hohendorf et al. were also typed as positive for the C*07 allele (20). HLA-C*07 is a ligand for activating and inhibiting killer immunoglobulin-like receptors (KIR) expressed by natural killer cells, and αβ and γδ T cells (40), and it has been suggested that a defect in KIR binding to HLA-C*07 may account for the association of this HLA allele with autoimmunity (31).

We also found that several RE patients shared HLA class II alleles suggesting a role for CD4^+^ T cells in disease. Six of the RE patients in our study cohort carried DQA1*04:01:01 and DQB1*04:02:01 alleles. Both alleles were less frequent in a reference population from a similar demographic suggesting that they may be associated with an increased risk of RE. HLA typing a much larger number of RE patients will be necessary to determine whether these alleles are associated with the disease. Several patients in the study cohort carried HLA class II alleles that have been linked to type 1 diabetes and MS.

Although far fewer CD4^+^ T cells than CD8^+^ T cells are found in affected brain parenchyma, CD4^+^ T cells are nevertheless present in perivascular spaces and leptomeninges (41, 42). CD4^+^ T cells play a multiplicity of roles in cellular immunity and T cell dependent antibody responses, and both CD4^+^ effector T cells and follicular CD4^+^ helper T cells have been implicated in autoimmune diseases (43, 44). Circulating autoantibodies against neuronal proteins have been detected in blood from some RE patients (45–48), and clonal expansion of immunoglobulin heavy chains in RE brain tissue has been reported (49). In one case report treatment of a RE patient with rituximab reduced seizures (5).

We have obtained an unbiased sample of the αβ T cell repertoire in 14 RE surgery cases by targeted sequencing genomic DNA from resected brain tissue and blood. We cannot rule out the possibility that immunosuppressive treatments prior to surgery may have impacted the T cell repertoires that we obtained. Calculating clonality scores revealed that the clonal composition of T cells in the brain was less diverse than in blood collected from the same patients at the time of the surgery. On average only about 15% of the brain αβ T cell repertoire was found in the blood. Some of T cells in the brain and the blood that expressed the same CDR3 amino acid sequence originated from different clonal lineages. We recently showed that the majority of T cells in resected brain tissue from seven RE surgeries expressed the resident memory T cell marker CD103 (50), thus many of T cell clones in the brain might not be expected to be part of the pool of circulating central memory and effector memory T cells (50). Resident T_RM_ cells develop at a site of infection during the effector stage of an immune response, and persist long after clearance of the pathogen (15). We have suggested that local reactivation of T_RM_ cells may explain why inflammation usually remains confined to one cerebral hemisphere in RE (50). In support of this idea, reactivation of T_RM_ cells appears to account for the reoccurrence of psoriatic lesions at the same sites on the skin (51).

In the brain only 18 TCRβ CDR3 sequences were found that were shared by three or more RE patients. All of these rare sequences were also detected in blood samples from FCD and TSC surgeries, and in almost half of the blood samples collected from a cohort of healthy individuals. The pathological significance of the low frequency T cell clones that express these public TCRβ CDR3 sequences is not clear. On the other hand 13 of the 31 most abundant TCRβ CDR3 sequences (defined as the top 5% of sequences in the 14 brain specimens) were also found in blood from normal donors. Nine of these public CDR3 sequences were the most frequently detected sequence in nine of the brain specimens. The public CDR3 nucleotide sequences were less divergent from germline than the abundant private CDR3 nucleotide sequences, which may reflect their specificity for common pathogens (35). In support of this idea, two of the abundant public TCRβ CDR3 sequences differ by a single amino acid from CDR3 sequences that are known to recognize an influenza virus epitope (Table 7) (36).

Structural or sequence similarity between pathogen peptides and self-peptides has been proposed as a cause of autoimmunity (52–54). In the Japanese population increased risk of RE has been linked to HLA-A*02:01, 24:02, 26:01, and HLA-B*46:01 (55). In the cohort of 24 RE cases that we analyzed, 41.7% of the patients carried HLA-A*02:01:01:01, and 20.8% of the patients carried HLA-A*24:02:01:01 (Table 1). Both HLA-A*02:01 and HLA-A*24:02 are known to bind peptides from influenza virus (38, 56). Takahashi et al have suggested that cytotoxic CD8^+^ T cells in RE brain may have arisen due to molecular mimicry (55). It may not be necessary to invoke molecular mimicry to explain the development of autoreactive T cells in as much as an infection can lead to the generation of T cells that express two TCRs, one directed against a foreign antigen, and a second TCR that recognizes a self-antigen (57). Alternatively, an inflammatory milieu created by an infection can cause the activation of weakly self-reactive bystander T cells that have escaped tolerance mechanisms (58), which may explain the presence of abundant private clones in RE brain. The two most abundant TCRβ CDR3 sequences identified in this study were patient specific and may also be disease-specific. Given the prevalence of these two TCRβ CDR3 sequences in the brain tissue sampled, it may be possible to identify the corresponding Vα and TCRα CDR3 by sequencing the same genomic DNA. This would allow us to reconstruct functional TCRs for antigen discovery and disease modeling (59, 60).

Thirteen of the abundant private TCRβ CDR3 sequences were present at a low frequency in the matched blood samples except in the case of RECP47. The αβ T cell repertoire in RECP47 blood was dominated by the same abundant CDR3 sequence found in the brain indicating that clones expressing this sequence had expanded in the periphery. Clonal expansion of circulating CD8^+^ T clones has been previously reported in several RE patients (20).

The co-occurrence of four different autoimmune diseases, Hashimoto thyroiditis, ulcerative colitis, Crohn disease, and systemic lupus erythematosus in four different RE patients has led to the suggestion that genes that predispose to autoimmunity are risk factors for RE (61). Our finding that some RE patients in our study cohort carry HLA alleles that are known to confer susceptibility to different autoimmune diseases strongly supports this idea.

## Ethics Statement

Human subjects were involved in this retrospective study, but only for the use of anonymized biological samples from surgeries to treat RE, FCD, and TSC that were donated to the Rare Epilepsies and Brain Disease Tissue Bank at UCLA. All of the surgical specimens used in this study were obtained under Institutional Review Board approval (UCLA IRB nos. 11-00030 and 13-001213). Patients were recruited free of coercion from pediatric epilepsy cases presenting to UCLA medical center and collaborating institutions for evaluation and diagnosis. Patients were consented at UCLA in compliance with UCLA IRB requirements or were consented according to collaborating institutions’ guidelines. In addition, all local regulatory requirements were adhered to.

## Author Contributions

SD and HW assisted with study design, prepared DNA libraries, and carried out sequencing; TG assisted with study design, and data analysis and interpretation; DH assisted with data analysis and interpretation; GM provided surgical specimens and support for the project; GO designed the study, analyzed the data and wrote the paper.

## Conflict of Interest Statement

The authors declare that the research was conducted in the absence of any commercial or financial relationships that could be construed as a potential conflict of interest.

## Footnotes

^1^www.imgt.org.

^2^www.r-project.org.

^3^www.mathwave.com.

^4^www.medcalc.org.

^5^http://bioinformatics.psb.ugent.be/webtools/Venn/.

^6^www.broadinstitute.org.

^7^www.allelefrequencies.net.
