# Supplementary material for: Shared HLA Class I and II Alleles and Clonally Restricted Public and Private Brain-Infiltrating αβ T Cells in a Cohort of Rasmussen Encephalitis Surgery Patients
Source: Front Immunol. 2016 Dec 19;7:608. doi: 10.3389/fimmu.2016.00608 (PMC5165278; doi:10.3389/fimmu.2016.00608)
Supplement: Supplementary file 1 [file Table_1.pdf]

Shared HLA Class I and II Alleles and Clonally Restricted Public and Private Brain-Infiltrating αβ T Cells in a Cohort of Rasmussen Encephalitis Surgery Patients.

Sugandha Dandekar, Hemani Wijesuriya, Tim Geiger, David Hamm, Gary W. Mathern, Geoffrey C. Owens*

***Correspondence:** Geoffrey C. Owens: geoffreyowens@mednet.ucla.edu

Supplementary Table 1: HLA typing of RE cases

	DPA1		DPB1		DQA1		DQB1		DRB1		HLA A		HLA B		HLA C	
Case ID	Allele 1	Allele 2	Allele 1	Allele 2	Allele 1	Allele 2	Allele 1	Allele 2	Allele 1	Allele 2	Allele 1	Allele 2	Allele 1	Allele 2	Allele 1	Allele 2
RECP02	DPA1*02:01:02	DPA1*02:02:02	DPB1*01:01:01	DPB1*01:01:01	DQA1*02:01	DQA1*05:01:01:01	DQB1*02:01:01	DQB1*02:02:01:01	DRB1*03:01:01:01	DRB1*07:01:01:01	A*02:01:01:01	A*02:01:01:01	B*44:02:01:01	B*44:02:01:01	C*05:01:01:01	C*07:04:01
RECP03	DPA1*01:03:01:01	DPA1*01:03:01:01	DPB1*04:01:01:01	DPB1*06:01	DQA1*03:01:01	DQA1*05:03	DQB1*03:01:01:01	DQB1*03:02:01	DRB1*04:04:01	DRB1*14:02:01	A*03:01:01:01	A*32:01:01	B*44:02:01:01	B*40:02:01	C*03:04:01:01	C*05:01:01:01
RECP12	DPA1*02:02:02	DPA1*03:01	DPB1*01:01:01	DPB1*105:01	DQA1*02:01	DQA1*04:01:01	DQB1*02:02:01	DQB1*04:02:01	DRB1*03:02:01	DRB1*07:01:01:01	A*03:01:01:01	A*33:01:01	B*07:02:01	B*27:03	C*07:02:01:01	C*02:02:02:01
RECP16	DPA1*01:03:01:01	DPA1*01:03:01:01	DPB1*04:01:01:01	DPB1*04:02:01:01	DQA1*01:01:02	DQA1*05:03	DQB1*02:02:01	DQB1*04:02:01	DRB1*01:02:01	DRB1*14:06:01	A*03:01:01:01	A*24:02:01:01	B*14:02:01:01	B*39:06:02	C*07:02:01:01	C*08:02:01:01
RECP26	DPA1*01:03:01:01	DPA1*01:03:01:01	DPB1*04:01:01:01	DPB1*04:01:01:01	DQA1*01:02:01:01	DQA1*05:05:01:01	DQB1*06:02:01	DQB1*03:01:01:01	DRB1*15:01:01:01	DRB1*01:03	A*03:01:01:01	A*24:02:01:01	B*07:02:01	B*27:05:02	C*07:02:01:01	C*01:02:01
RECP27	DPA1*01:03:01:01	DPA1*01:03:01:01	DPB1*04:01:01:01	DPB1*03:01:01	DQA1*04:01:01	DQA1*01:02:01:01	DQB1*06:02:02	DQB1*04:02:01	DRB1*15:01:01:01	DRB1*08:01:01	A*02:01:01:01	A*03:01:01:01	B*07:02:01	B*39:06:02	C*07:02:01:01	C*07:02:01:01
RECP30	DPA1*01:03:01:01	DPA1*01:03:01:01	DPB1*04:01:01:01	DPB1*02:01:02	DQA1*03:03:01	DQA1*05:03	DQB1*03:01:01:01	DQB1*03:01:01:01	DRB1*14:02:01	DRB1*04:01:01	A*02:01:01:01	A*01:01:01:01	B*35:01:04	B*44:02:07	C*04:01:01:01	C*05:01:01:01
RECP31	DPA1*01:03:01:01	DPA1*01:03:01:01	DPB1*04:01:01:01	DPB1*03:01:01	DQA1*03:01:01	DQA1*01:02:01:01	DQB1*06:04:01	DQB1*03:02:01	DRB1*13:02:01	DRB1*04:01:01	A*33:03:01	A*01:01:01:01	B*15:01:01:01	B*40:01:02	C*03:04:27	C*03:02:02:01
RECP32	DPA1*01:03:01:01	DPA1*02:02:02	DPB1*01:01:01	DPB1*18:01	DQA1*04:01:01	DQA1*05:01:01:01	DQB1*02:01:01	DQB1*04:02:01	DRB1*03:01:01:01	DRB1*03:02:01	A*33:03:01	A*33:03:01	B*07:02:01	B*44:03:01:01	C*04:01:01:01	C*03:02:02:01
RECP33	DPA1*01:03:01:01	DPA1*02:01:08	DPB1*01:01:01	DPB1*03:01:01	DQA1*05:05:01:01	DQA1*05:01:01:01	DQB1*02:01:01	DQB1*03:19	DRB1*03:01:01:01	DRB1*11:02:01	A*02:01:01:01	A*02:02:01:01	B*08:01:01	B*53:01:01	C*04:01:01:01	C*07:01:01:01
RECP34	DPA1*01:03:01:01	DPA1*02:02:02	DPB1*04:01:01:01	DPB1*03:01:01	DQA1*02:01	DQA1*01:03:01:01	DQB1*02:02:01:01	DQB1*06:03:01	DRB1*13:01:01	DRB1*07:01:01:01	A*03:01:01:01	A*24:02:01:01	B*07:02:01	B*13:02:01	C*07:02:01:01	C*06:02:01:01
RECP35	DPA1*01:03:01:01	DPA1*01:03:01:01	DPB1*04:01:01:01	DPB1*105:01	DQA1*02:01	DQA1*03:01:01	DQB1*03:02:01	DQB1*03:03:02:01	DRB1*04:03:01	DRB1*07:01:01:01	A*29:02:01:01	A*68:03:01	B*35:43:01	B*44:03:01:01	C*04:01:01:01	C*01:02:01
RECP37	DPA1*01:03:01:01	DPA1*01:03:01:01	DPB1*04:01:01:01	DPB1*03:01:01	DQA1*01:03:01:01	DQA1*05:01:01:01	DQB1*02:01:01	DQB1*06:03:01	DRB1*03:01:01:01	DRB1*13:01:01	A*02:01:01:01	A*24:03:01	B*44:02:01:01	B*35:01:01:01	C*04:01:01:01	C*05:01:01:01
RECP38	DPA1*01:03:01:01	DPA1*01:03:01:01	DPB1*04:02:01:01	DPB1*02:01:02	DQA1*02:01	DQA1*05:05:01:01	DQB1*02:02:01	DQB1*03:01:01:01	DRB1*11:04:01	DRB1*07:01:01:01	A*02:01:01:01	A*24:02:01:01	B*35:02:01	B*50:01:01	C*04:01:01:01	C*06:02:01:01
RECP39	DPA1*01:03:01:01	DPA1*02:06	DPB1*04:01:01:01	DPB1*05:01:01	DQA1*03:02	DQA1*01:02:01:01	DQB1*06:02:01	DQB1*03:03:02:01	DRB1*15:01:01:01	DRB1*09:01:02	A*02:01:01:01	A*03:01:01:01	B*07:02:01	B*07:02:01	C*07:02:01:01	C*07:02:01:01
RECP40	DPA1*01:03:01:01	DPA1*01:03:01:01	DPB1*04:01:01:01	DPB1*04:01:01:01	DQA1*03:01:01	DQA1*01:02:02	DQB1*05:02:01	DQB1*03:02:01	DRB1*16:01:01	DRB1*16:15	A*68:01:02:01	A*68:01:02:01	B*07:02:01	B*40:01:02	C*07:02:01:01	C*03:04:01:01
RECP42	DPA1*01:03:01:01	DPA1*01:03:01:01	DPB1*04:01:01:01	DPB1*04:01:01:01	DQA1*02:01	DQA1*01:02:01:01	DQB1*06:02:01	DQB1*02:02:01:01	DRB1*15:01:01:01	DRB1*07:01:01:01	A*23:01:01	A*11:01:01:01	B*07:02:01	B*07:05:01	C*07:02:01:01	C*15:05:02
RECP43	DPA1*01:03:01:01	DPA1*01:03:01:01	DPB1*04:02:01:01	DPB1*03:01:01	DQA1*03:01:01	DQA1*05:05:01:01	DQB1*03:01:01:01	DQB1*03:02:01	DRB1*11:04:01	DRB1*04:03:01	A*29:02:01:01	A*11:01:01:01	B*18:01:01:01	B*44:03:01:01	C*07:01:01:01	C*16:01:01:01
RECP45	DPA1*01:03:01:01	DPA1*01:03:01:01	DPB1*04:01:01:01	DPB1*02:01:02	DQA1*04:01:01	DQA1*01:03:01:01	DQB1*06:03:01	DQB1*04:02:01	DRB1*13:01:01	DRB1*08:01:01	A*02:01:01:01	A*24:02:01:01	B*15:01:01:01	B*39:06:02	C*07:02:01:01	C*03:03:01
RECP46	DPA1*01:03:01:01	DPA1*01:03:01:01	DPB1*04:02:01:01	DPB1*04:02:01:01	DQA1*04:01:01	DQA1*04:01:01	DQB1*04:02:01	DQB1*04:02:01	DRB1*08:02:01	DRB1*08:02:01	A*02:01:01:01	A*02:01:01:01	B*40:74	B*48:13	C*08:01:01	C*15:02:01:01
RECP47	DPA1*02:01:08	DPA1*03:01	DPB1*105:01	DPB1*01:01:01	DQA1*04:01:01	DQA1*05:05:01:01	DQB1*03:19	DQB1*04:02:01	DRB1*03:29	DRB1*11:48	A*02:01:01:01	A*80:01:01:01	B*35:01:01:01	B*78:01:01	C*04:01:09	C*16:01:06
RECP48	DPA1*01:03:01:01	DPA1*01:03:01:01	DPB1*04:02:01:01	DPB1*03:01:01	DQA1*03:01:01	DQA1*05:05:01:01	DQB1*03:01:01:01	DQB1*03:02:01	DRB1*11:04:01	DRB1*04:03:01	A*29:02:01:01	A*11:01:01:01	B*18:01:01:01	B*44:03:01:01	C*07:01:01:01	C*16:01:01:01
RECP49	DPA1*01:03:01:01	DPA1*02:01:01	DPB1*04:01:01:01	DPB1*05:01:01	DQA1*01:02:01:01	DQA1*01:02:01:01	DQB1*06:02:01	DQB1*06:09:01	DRB1*15:01:01:01	DRB1*13:02:01	A*29:02:01:01	A*03:01:01:01	B*07:02:01	B*14:02:01:01	C*07:02:01:01	C*08:02:01:01
RECP50	DPA1*01:03:01:01	DPA1*02:01:01	DPB1*04:01:01:01	DPB1*11:01:01	DQA1*02:01	DQA1*01:02:01:01	DQB1*06:02:01	DQB1*02:02:01	DRB1*15:01:01:01	DRB1*07:01:01:01	A*29:02:01:01	A*68:01:02:01	B*15:01:01:01	B*44:03:01:01	C*03:04:01:01	C*16:01:01:01

RECP, RE Children's Project
