# Supplementary material for: Shared HLA Class I and II Alleles and Clonally Restricted Public and Private Brain-Infiltrating αβ T Cells in a Cohort of Rasmussen Encephalitis Surgery Patients
Source: Front Immunol. 2016 Dec 19;7:608. doi: 10.3389/fimmu.2016.00608 (PMC5165278; doi:10.3389/fimmu.2016.00608)
Supplement: Supplementary file 2 [file Table_2.pdf]

Shared HLA Class I and II Alleles and Clonally Restricted Public and Private Brain-Infiltrating αβ T Cells in a Cohort of Rasmussen Encephalitis Surgery Patients.

Sugandha Dandekar, Hemani Wijesuriya, Tim Geiger, David Hamm, Gary W. Mathern, Geoffrey C. Owens*

***Correspondence:** Geoffrey C. Owens: geoffreyowens@mednet.ucla.edu

Supplementary Table 2: Nucleotide sequences of top CDR3s in RE brain specimens with multiple TRBV gene usage.

Case ID	CDR3 (amino acid)	CDR3 (nucleotide)	Vβ gene	Freq.
RECP33 brain	CAISESNYGYTF	TGTGCCATCAGTGAATCTAACTATGGCTACACCTTCGGTTC	TRBV6	0.65
		TGTGCCATCAGTGAATCTAACTATGGCTACACCTTCGGTTC	TRBV10	99.35
	CASSLLVVESELHTGELFF	TGTTGGTGGTGGAGTCGGAGTTGCACACCGGGGAGCTGTTTTTT	TRBV4	0.18
		TGTTGGTGGTGGAGTCGGAGTTGCACACCGGGGAGCTGTTTTTT	TRBV5	99.28
		TGTTGGTGGTGGAGTCGGAGTTGCACACCGGGGAGCTGTTTTTG	TRBV5	0.54
	CASSKTSGPDNEQFF	TGTGCCAGCAGCAAGACTAGCGGGCCTGACAATGAGCAGTTCTTC	TRBV7	98.66
		TGTGCCAGCAGCAAGACTAGCGGGCCTGACAATGAGCAGTTCTTC	TRBV11	1.34
RECP34 brain	CASAEEWSSYNSPLHF	TGTGCCAGCGCCGAGGAGTGGAGCTCCTATAATTCACCCCTCCACTTT	TRBV6	99.89
		TGTGCCAGCGCCGAGGAGTGGAGCTCCTATAATTCACCCCTCCACTTT	TRBV10	0.11
	CASSQDTPGQFYEQYF	TGCGCCAGCAGCCAAGACACTCCGGGACAGTTCTACGAGCAGTACTTC	TRBV1	0.15
		TGCGCCAGCAGCCAAGACACTCCGGGACAGTTCTACGAGCAGTACTTC	TRBV4	98.78
		TGTGCCAGCAGCCAAGACACTCCGGGACAGTTCTACGAGCAGTACTTC	TRBV5	0.46
		TGCGCCAGCAGCCAAGACACTCCGGGACAGTTCTACGAGCAGTACTTC	TRBV7	0.46
		TGCGCCAGCAGCCAAGACACTCCGGGACAGTTCTACGAGCAGTACTTC	TRBV13	0.15
RECP35 brain	CASSLRGTGNTEAFF	TGTGCCAGCAGCTTAAGGGGAACGGGGAACACTGAAGCTTTCTTT	TRBV2	0.2
		TGTGCCAGCAGCTTAAGGGGAACGGGGAACACTGAAGCTTTCTTT	TRBV4	0.39
		TGTGCCAGCAGCTTAAGGGGAACGGGGAACACTGAAGCTTTCTTT	TRBV7	0.2
		TGTGCCAGCAGCTTAAGGGGAACGGGGAACACTGAAGCTTTCTTT	TRBV12	0.39
		TGTGCCAGCAGCTTAAGGGGAACGGGGAACACTGAAGCTTTCTTT	TRBV19	0.59
		TGTGCCAGCAGCTTAAGGGGAACGGGGAACACTGAAGCTTTCTTT	TRBV27	0.39
		TGTGCCAGCAGCTTAAGGGGAACGGGGAACACTGAAGCTTTCTTT	TRBV28	0.39
		TGTGCCAGCAGCTTAAGGGGAACGGGGAACACTGAAGCTTTCTTT	TRBV30	97.45
	CASGPGGPSTGELFF	TGTGCCAGCGGCCCAGGGGGGCCGAGCACCGGGGAGCTGTTTTTT	TRBV12	0.22
		TGTGCCAGCGGCCCAGGGGGGCCGAGCACCGGGGAGCTGTTTTTT	TRBV13	99.78
	CASSTSSTDTQYF	TGTGCCAGCAGTACATCTAGCACAGATACGCAGTATTTT	TRBV2	99.69
		TGTGCCAGCAGTACATCTAGCACAGATACGCAGTATTTT	TRBV6	0.31
RECP37 brain	CASSQDPQGALNEQFF	TGCGCCAGCAGCCAAGATCCCCAGGGGGCGCTGAATGAGCAGTTCTTC	TRBV4	99.28
		TGCGCCAGCAGCCAAGATCCCCAGGGGGCGCTGAATGAGCAGTTCTTC	TRBV5	0.36
		TGCGCCAGCAGCCAAGATCCCCAGGGGGCGCTGAATGAGCAGTTCTTC	TRBV7	0.09
		TGCGCCAGCAGCCAAGATCCCCAGGGGGCGCTGAATGAGCAGTTCTTC	TRBV11	0.18
		TGTGCCAGCAGCCAAGATCCCCAGGGGGCGCTGAATGAGCAGTTCTTC	TRBV11	0.09
RECP40 brain	CASSVAYEQYF	TGTGCCAGCAGTGTAGCGTACGAGCAGTACTTC	TRBV2	0.04
		TGTGCCAGCAGTGTCGCCTACGAGCAGTACTTC	TRBV3	0.2
		TGTGCCAGCAGTGTAGCGTACGAGCAGTACTTC	TRBV6	64.08
		TGTGCCAGCAGTGTCGCCTACGAGCAGTACTTC	TRBV6	34.86
		TGTGCCAGCAGCGTGGCCTACGAGCAGTACTTC	TRBV6	0.4
		TGTGCCAGCAGTGTAGCGTACGAGCAGTACTTC	TRBV10	0.15
		TGTGCCAGCAGTGTAGCGTACGAGCAGTACTTC	TRBV12	0.15
		TGTGCCAGCAGTGTCGCCTACGAGCAGTACTTC	TRBV25	0.04
		TGTGCCAGCAGTGTCGCGTACGAGCAGTACTTC	TRBV27	0.08
	CASAVAYEQYF	TGTGCCAGCGCAGTTGCCTACGAGCAGTACTTC	TRBV3	0.09
		TGTGCCAGCGCAGTTGCCTACGAGCAGTACTTC	TRBV6	99.67
		TGTGCCAGCGCAGTTGCCTACGAGCAGTACTTC	TRBV10	0.1
		TGTGCCAGCGCAGTTGCCTACGAGCAGTACTTC	TRBV12	0.09
		TGTGCCAGCGCAGTTGCCTACGAGCAGTACTTC	TRBV27	0.05
RECP42 brain	CASSVDHRAGKPYEQYF	TGTGCCAGTAGTGTGGATCACAGGGCAGGGAAACCCTACGAGCAGTACTTC	TRBV5	0.004
		TGTGCCAGTAGTGTGGATCACAGGGCAGGGAAACCCTACGAGCAGTACTTC	TRBV19	99.971
		TGTGCCAGTAGTGTGGATCACAGGGAAGGGAAACCCTACGAGCAGTACTTC	TRBV19	0.025
	CATSVTTGGYTEAFF	TGTGCCACCAGTGTCACAACAGGGGGTTACACTGAAGCTTTCTTT	TRBV12	0.02
		TGTGCCACCAGTGTCACAACAGGGGGTTACACTGAAGCTTTCTTT	TRBV24	99.98
	CASSGGSTDTQYF	TGTGCCAGTAGCGGGGGGAGCACAGATACGCAGTATTTT	TRBV4	0.02
		TGTGCCAGTAGCGGGGGGAGCACAGATACGCAGTATTTT	TRBV19	99.98
RECP43 brain	CASSLQDRGPGGEQYV	TGTGCCAGCAGCCTACAGGACAGGGGGCCCGGTGGCGAGCAGTACGTC	TRBV7	3.33
		TGTGCCAGCAGCCTACAGGACAGGGGGCCCGGTGGCGAGCAGTACGTC	TCRBV11	96.67
RECP45 brain	CASSLRNYDDRVGYYEQYF	TGTGCCAGCAGTTTACGAAACTACGACGACAGGGTGGGTTACTACGAGCAGTACTTC	TRBV6	0.08
		TGTGCCAGCAGTTTACGAAACTACGACGACAGGGTGGGTTACTACGAGCAGTACTTC	TRBV12	0.04
		TGTGCCAGCAGTTTACGAAACTACGACGACAGGGTGGGTTACTACGAGCAGTACTTC	TRBV27	99.8
		TGTGCCAGCAGTTTACGAAACTACGACGACAGGGTGGGTTACTACGAGCAGTACTTC	TRBV28	0.08
	CASSLGTGDRSNQPQHF	TGTGCCAGCAGTTTAGGGACAGGGGACAGGAGCAATCAGCCCCAGCATTTT	TRBV6	0.02
		TGTGCCAGCAGTTTAGGGACAGGGGACAGGAGCAATCAGCCCCAGCATTTT	TRBV28	99.98
RECP49 brain	CASQLGAATGYTF	TGTGCCAGCCAGCTGGGGGCCGCGACTGGCTACACCTTC	TRBV4	0.04
		TGTGCCAGCCAGCTGGGGGCCGCGACTGGCTACACCTTC	TRBV5	0.06
		TGTGCCAGCCAGCTGGGGGCCGCGACTGGCTACACCTTC	TRBV7	0.08
		TGTGCCAGCCAGCTGGGGGCCGCGACTGGCTACACCTTC	TCRBV11	99.82
RECP50	CASSLSSFQETQYF	TGTGCCAGCAGCTTATCTTCATTCCAAGAGACCCAGTACTTC	TRBV7	99.78
		TGTGCCAGCAGCTTATCTTCATTCCAAGAGACCCAGTACTTC	TRBV11	0.22
