# Supplementary material for: Shared HLA Class I and II Alleles and Clonally Restricted Public and Private Brain-Infiltrating αβ T Cells in a Cohort of Rasmussen Encephalitis Surgery Patients
Source: Front Immunol. 2016 Dec 19;7:608. doi: 10.3389/fimmu.2016.00608 (PMC5165278; doi:10.3389/fimmu.2016.00608)
Supplement: Supplementary file 3 [file Data_Sheet_1.pdf]

Shared HLA Class I and II Alleles and Clonally Restricted Public and Private Brain-Infiltrating αβ T Cells in a Cohort of Rasmussen Encephalitis Surgery Patients.

Sugandha Dandekar, Hemani Wijesuriya, Tim Geiger, David Hamm, Gary W. Mathern, Geoffrey C. Owens*

***Correspondence:** Geoffrey C. Owens: geoffreyowens@mednet.ucla.edu

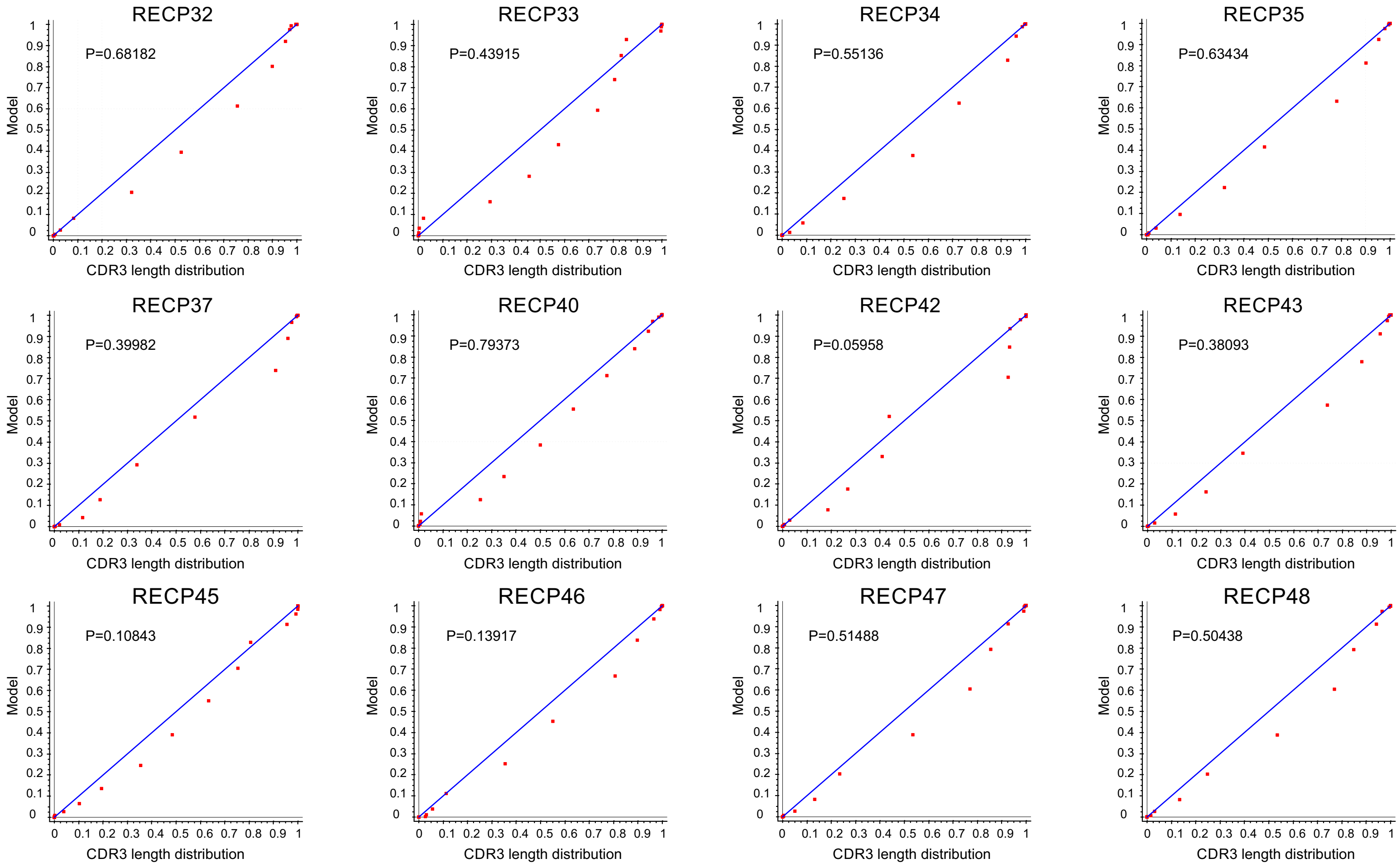

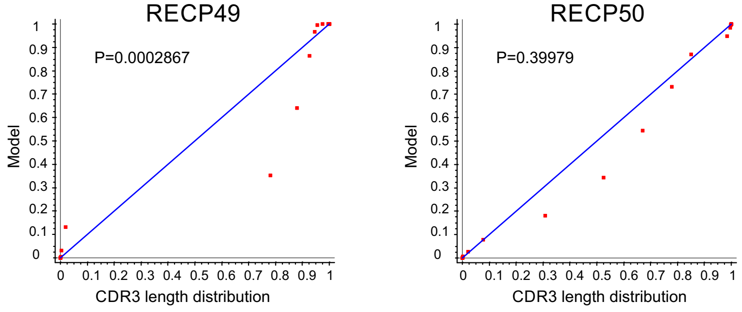

Supplementary Figure 1: Normal probability plots to determine whether CDR3 lengths are normally distributed. The cumulative distribution function for CDR3 lengths is plotted against the cumulative distribution function for a normal distribution. A significant deviation from the straight line indicates that the CDR3 lengths are not normally distributed. The Kolmogorov-Smirnov test was used to test for significance.
