# Supplementary material for: Shared HLA Class I and II Alleles and Clonally Restricted Public and Private Brain-Infiltrating αβ T Cells in a Cohort of Rasmussen Encephalitis Surgery Patients
Source: Front Immunol. 2016 Dec 19;7:608. doi: 10.3389/fimmu.2016.00608 (PMC5165278; doi:10.3389/fimmu.2016.00608)
Supplement: Supplementary file 4 [file Data_Sheet_2.pdf]

Shared HLA Class I and II Alleles and Clonally Restricted Public and Private Brain-Infiltrating αβ T Cells in a Cohort of Rasmussen Encephalitis Surgery Patients.

Sugandha Dandekar, Hemani Wijesuriya, Tim Geiger, David Hamm, Gary W. Mathern, Geoffrey C. Owens*

***Correspondence:** Geoffrey C. Owens: geoffreyowens@mednet.ucla.edu

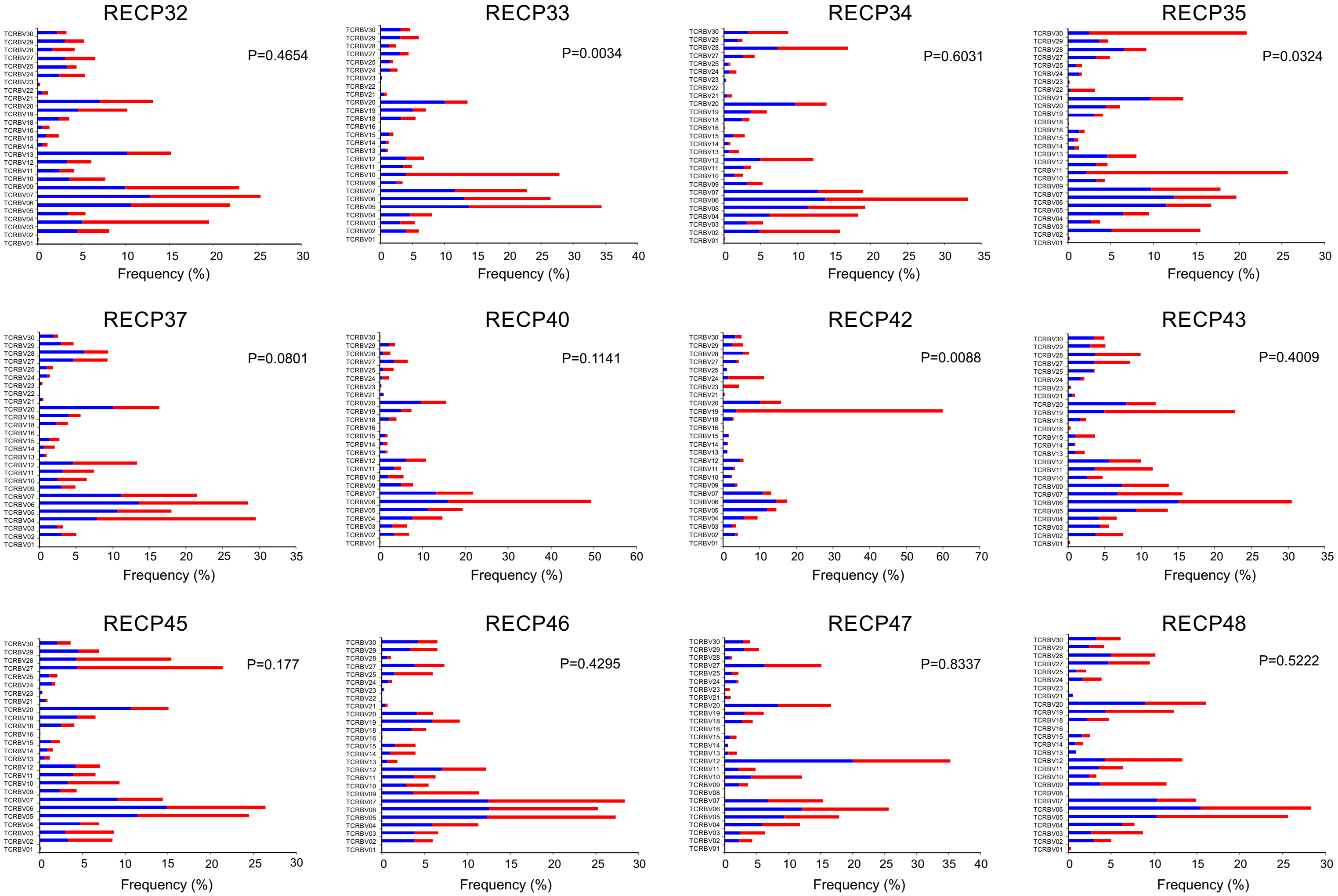

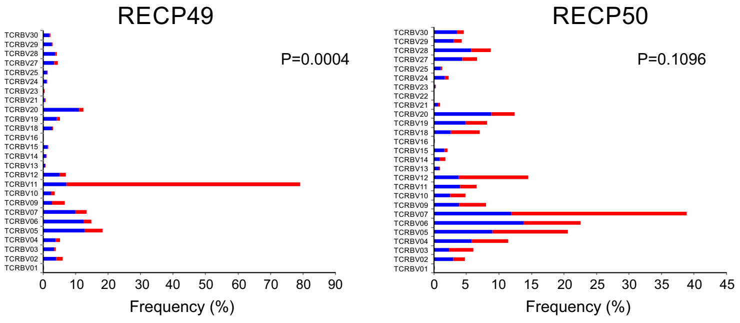

Supplementary Figure 2: Vβ gene usage in fourteen Rasmussen encephalitis cases. Bar graphs showing the frequency of Vβ genes in each Rasmussen encephalitis (RE) brain and blood specimen. For each Vβ gene the portion of the bar in blue shows the frequency in the blood and the portion of the bar in red corresponds to the frequency in the brain. The Wilcoxon signed-rank test was used to compare Vβ gene frequencies in the brain and blood of each RE case.
