# Supplementary material for: Shared HLA Class I and II Alleles and Clonally Restricted Public and Private Brain-Infiltrating αβ T Cells in a Cohort of Rasmussen Encephalitis Surgery Patients
Source: Front Immunol. 2016 Dec 19;7:608. doi: 10.3389/fimmu.2016.00608 (PMC5165278; doi:10.3389/fimmu.2016.00608)
Supplement: Supplementary file 5 [file Data_Sheet_3.pdf]

Shared HLA Class I and II Alleles and Clonally Restricted Public and Private Brain-Infiltrating αβ T Cells in a Cohort of Rasmussen Encephalitis Surgery Patients.

Sugandha Dandekar, Hemani Wijesuriya, Tim Geiger, David Hamm, Gary W. Mathern, Geoffrey C. Owens*

***Correspondence:** Geoffrey C. Owens: geoffreyowens@mednet.ucla.edu

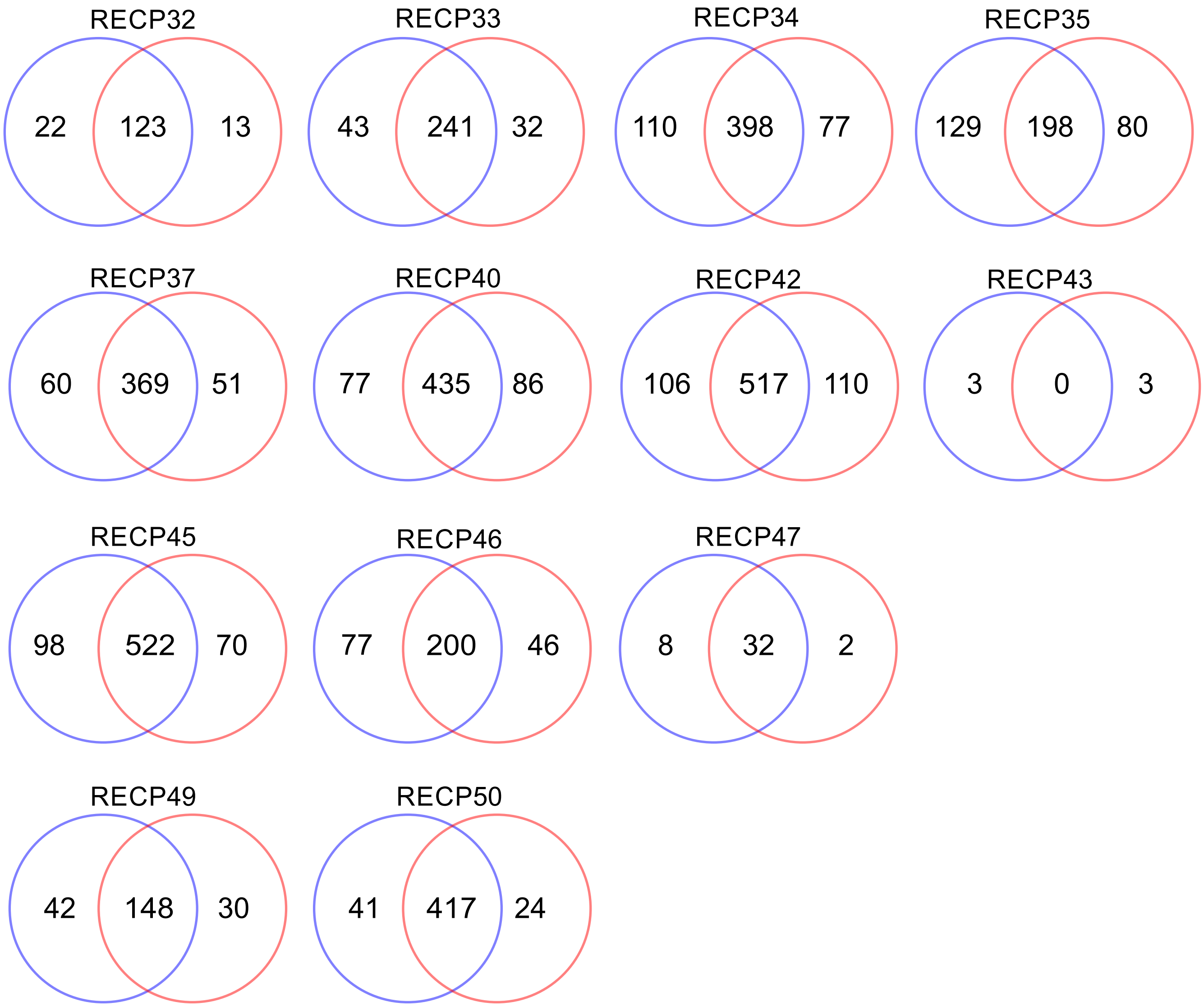

Case ID	CDR3 (amino acid)	CDR3 (nucleotide)	Vβ gene	Freq. (%)
RECP43 brain	CASSAGNTGELFF	TGTGCCAGCAGTGCAGGGAACACCGGGGAGCTGTTTTTT	TRBV06	0.17
RECP43 blood		TGTGCCAGCAGTGCGGGGAACACCGGGGAGCTGTTTTTT	TRBV06	0.003
RECP43 brain	CASSLQGTEAFF	TGTGCCAGCAGCTTACAGGGAACTGAAGCTTTCTTT	TRBV07	0.17
RECP43 blood		TGTGCCAGCAGTTTACAGGGCACTGAAGCTTTCTTT	TRBV28	0.003
RECP43 brain	CASSPQGHYGYTF	TGTGCCAGCAGCCCACAGGGCCACTATGGCTACACCTTC	TRBV12	0.17
RECP43 blood		TGTGCCAGCTCACCACAGGGGCATTATGGCTACACCTTC	TRBV18	0.003

Supplementary Figure 3: Different T cell clones with the same specificity are present in the brain and blood from Rasmussen encephalitis patients. For each case the number of nucleotide sequences that encode CDR3 sequences that are shared between the brain and the blood were compiled and displayed as a Venn diagram. The nucleotide sequences of the three CDR3s shared by RECP43 brain and blood are presented in the table; differences are shown in red.
